# Dealing with highly skewed hospital length of stay distributions: The use of Gamma mixture models to study delivery hospitalizations

**DOI:** 10.1371/journal.pone.0231825

**Published:** 2020-04-20

**Authors:** Eva Williford, Valerie Haley, Louise-Anne McNutt, Victoria Lazariu

**Affiliations:** 1 Department of Epidemiology and Biostatistics, University at Albany, State University of New York, Albany, New York, United States of America; 2 Institute for Health and the Environment, University at Albany, State University of New York, Albany, New York, United States of America; Kilimanjaro Christian Medical University College, UNITED REPUBLIC OF TANZANIA

## Abstract

The increased focus on addressing severe maternal morbidity and maternal mortality has led to studies investigating patient and hospital characteristics associated with longer hospital stays. Length of stay (LOS) for delivery hospitalizations has a strongly skewed distribution with the vast majority of LOS lasting two to three days in the United States. Prior studies typically focused on common LOSs and dealt with the long LOS distribution tail in ways to fit conventional statistical analyses (e.g., log transformation, trimming). This study demonstrates the use of Gamma mixture models to analyze the skewed LOS distribution. Gamma mixture models are flexible and, do not require data transformation or removal of outliers to accommodate many outcome distribution shapes, these models allow for the analysis of patients staying in the hospital for a longer time, which often includes those women experiencing worse outcomes. Random effects are included in the model to account for patients being treated within the same hospitals. Further, the role and influence of differing placements of covariates on the results is discussed in the context of distinct model specifications of the Gamma mixture regression model. The application of these models shows that they are robust to the placement of covariates and random effects. Using New York State data, the models showed that longer LOS for childbirth hospitalizations were more common in hospitals designated to accept more complicated deliveries, across hospital types, and among Black women. Primary insurance also was associated with LOS. Substantial variation between hospitals suggests the need to investigate protocols to standardize evidence-based medical care.

## Introduction

The United States (US) spends more per person on health care than any other nation, yet still performs poorly on key population health measures [1]. Childbirth is an unusually dangerous experience in young women’s lives. Recently in the US, attention has focused on the unacceptably high maternal mortality rate. Forty-six countries, including all of Western Europe, Canada, Australia, and Japan have substantially lower maternal mortality rates than the US (3–9 versus 14 deaths per 100,000 deliveries, respectively) [2].

For every maternal death many more women are experiencing severe maternal morbidity (SMM). SMM is defined as an injury, illness or condition that could lead to maternal death or disability. A group of indicators are utilized to measure SMM, including direct measures (e.g., cardiac arrest, stroke) and indirect measures (e.g., blood transfusions, intensive care unit admission). A subset of these indicators apply only to women with a longer LOS [3,4]. SMM has been increasing in the US, likely due to the older ages of women having children, the obesity epidemic and, associated with both these factors, increases in comorbidities [3,4]. Annually, more than 50,000 women have a SMM indicator during their delivery hospitalization, resulting in serious medical complications and extended hospital stays [2–4]. To reduce SMM and maternal mortality, research and intervention development efforts are focused on improvement of maternal health and health care.

Childbirth is the most common reason for hospitalization in the US with approximately four million deliveries annually. The cost of maternal hospitalizations totaled approximately $18.9 billion in the US for 2014 [5]. Federal regulations require health insurance plans to provide coverage for a minimum hospital stay of 48 hours following a vaginal delivery and 96 hours following a Cesarean delivery [6]. The average vaginal delivery without complications costs $3,490 and the average Cesarean delivery without complications costs $5,611 in the US [7]. Costs increase as complications arise during delivery or when hospital stays are extended.

To improve the quality of maternal care and allocation of healthcare resources, it is important to determine patient and hospital characteristics that influence the variation of LOS. Women may experience a varying number and severity of complications during their delivery, and a longer hospital stay may be necessary. The patients with longer LOS represent an important group that may have experienced SMM. Understanding LOS and balancing health care costs and quality of care in defining appropriate LOS are important public health focuses [2,8]. Hospital administrators can benefit from the use of better predictive models to assist with planning and resource allocation for deliveries. In this study, we present finite mixture models as a modeling technique to understand the influence of patient and hospital characteristics for all patients’ delivery LOS.

### Statistical literature review

**Fig 1:**
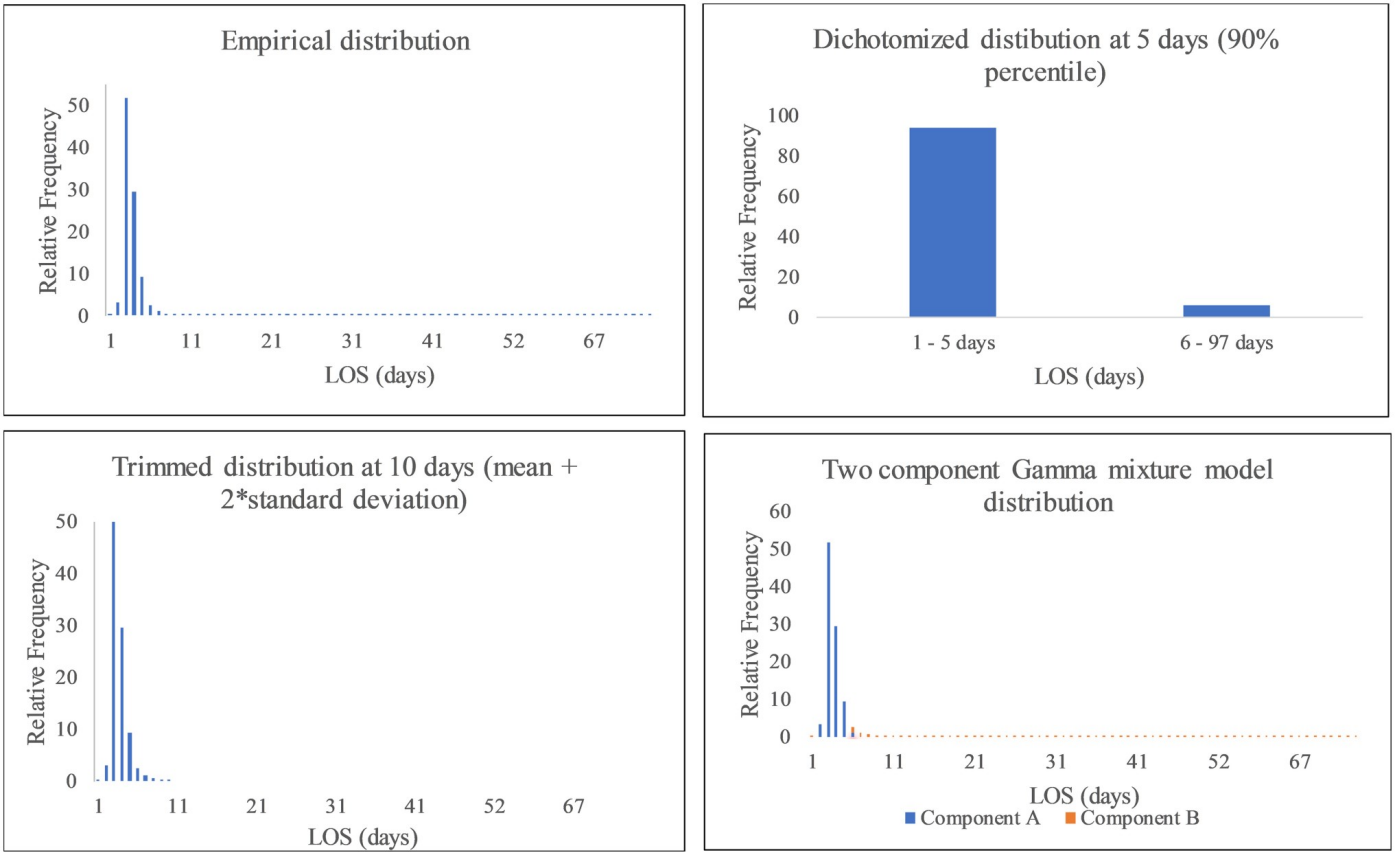
Comparing methods for length of stay (LOS) distributions for New York City Cesarean deliveries.

The empirical distribution of LOS for delivery hospitalizations is typically right skewed, plurimodal, and contains outliers. These distributional properties pose challenges in statistical analysis. Modeling the LOS distribution is not amenable to conventional parametric models (e.g., multiple linear regression) as it often violates normality and independence assumptions. Thus, various methods have been proposed to model the LOS distribution (Fig 1). Lee, et al. [9], Lee, et al. [10], Wang, et al. [11], Lee et al. [12], Leung, et al. [13], Cots, et al. [14], Freitas, et al. [15], and Marazzi, et al. [16] used trimming methods (e.g., mean plus two standard deviations) on the LOS to identify outliers and build models using the trimmed data. However, trimming points are defined without theoretical support. Additionally, some distributional characteristics of LOS are ignored when trimming methods are applied. Lee, et al. [17], Faddy, et al. [18], Ng, et al. [19], Xiao, et al. [20], and Yau, et al. [21] used logarithm transformations on LOS to attain normality. Unfortunately, transformation for LOS is not always appropriate as the data may not be well approximated by the log-normal distribution, thus applying the transformation does not sufficiently reduce skewness [16].

Finite mixture models may be better alternatives to analyze LOS compared to trimming methods or transformations since these models account for the distributional characteristics of LOS and incorporate all data into the models. A finite mixture model is a weighted sum of distributions and does not require transformation or defined trim points. A variety of distributions such as Gamma, Weibull or Normal can be used. Further information on finite mixture model theory can be found in McLachlan and Peel [22].

Delivery hospitalizations may be conceptualized as a combination of distributions for subpopulations of women with differences in demographics and clinical characteristics (e.g., comorbidities, complications) and delivery methods (e.g., vaginal vs Cesarean section, hospital protocols). Finite mixture models distinguish subpopulations of women’s LOS by the component distributions that create the overall distribution.

The current literature focuses on applying the methodology of finite mixture models to analyzing LOS and developing algorithms for model estimation [17,19,20,23–28]. Finite mixture models can incorporate patient level and hospital level characteristics (covariates) that influence LOS for different subpopulations (components). However, there is a research gap on the placement and selection of covariates in finite mixture model analysis. The aim of this paper is to explore the role of covariates in finite mixture regression model specifications and their impact on the resulting components. This new work enhances the application of finite mixture models for understanding the influence of covariates on hospital LOS for delivery.

## Methods

In this study a finite mixture distribution is used to model delivery LOS distributions. Patient level (e.g., age) and hospital level (e.g., teaching status) characteristics are incorporated in the Gamma mixture regression models. The addition of patient and hospital characteristics refines the LOS predictions. The location of the covariates and their influence on model results are evaluated. Gamma mixture models can be specified with patient and hospital characteristics in the mixing probabilities function and in the component density functions. In determining factors that influence LOS, the clustering of patients treated within the same hospitals needs to be modelled because differences in hospital practices and procedures may influence LOS. This clustering is accounted for using random hospital effects.

### Gamma mixture distribution

Gamma mixture models accommodate a wide range and high variability associated with the empirical distribution of delivery LOS. Mixtures of Gamma distributions can identify the subpopulations by breaking down the empirical distribution of LOS into a weighted sum of elementary components. Thus, Gamma mixture distributions are flexible and can model multiple subpopulations. Mixture models using other distributions such as Poisson have been used to study LOS [11]. The Gamma distribution is appropriate for modeling LOS given the flexibility in accommodating varying degrees of skewness. For example, the distribution of delivery LOS may be decomposed into multiple subpopulations with different LOS distributions.

Let *y*_*i*_ represent the LOS for the *i*^*th*^ woman, *i* = 1, …, *n*. The probability density function of *Y* with *c* components is defined as:

$$f(y_{i})=\sum_{j=1}^{c}\pi_{j}{(x_{i})f}_{j}\text{(}y_{i}\text{;}\theta_{j})$$

where, *π*_*j*_(*x*_*i*_) gives the proportion of individuals belonging to the *j*^*th*^ component ($\sum_{j=1}^{c}\pi_{j}(x_{i})=1$) and *f*_*j*_ (*y*_*i*_;*θ*_*j*_) denotes the *j*^*th*^ component density. This finite mixture model takes into account the variability within components and estimates the proportions of the components based on the LOS. In this paper, we use a Gamma mixture distribution to model the LOS. The Gamma probability density function is parameterized as follows for the *j*^*th*^ component,

$$f_{j}(y_{i};\theta_{j})=\frac{1}{y_{i}ℾ(v_{j})}{\left(\frac{v_{j}y_{i}}{\mu_{j}}\right)}^{v_{j}}e^{-\;\frac{v_{j}y_{i}}{\mu_{j}}}$$

with mean *μ*_*j*_ and shape parameter *v*_*j*_. The Gamma distribution is flexible for different degrees of skewness and is bounded by zero on the left.

### Gamma mixture regression models

Gamma mixture regression models, which are special cases of generalized linear models for finite mixtures, are fit to the empirical LOS distribution with patient and hospital characteristics. Gamma mixture models can be specified with covariates in the mixing probabilities function and in the component density functions. Thus, the identified covariates can be compared between the subpopulations (components).

In mixture models, mixing probabilities are assumed to be either constant or dependent on covariates. Constant mixing probabilities assume all individuals have equal prior probabilities of being a member of a given component. Mixing probabilities that depend on covariates are modeled using multinomial logistic regression that allows the covariates to influence component membership. The probability of the *i*^*th*^ individual belonging to the *j*^*th*^ component is denoted as:

$$\pi_{j}(x_{i})=\frac{exp\;(\sum_{l=0}^{p}\alpha_{jl}x_{il})}{1+\;\sum_{h=1}^{c-1}exp\;(\sum_{l=0}^{p}\alpha_{hl}x_{il})}$$

where the *x*_*i*_ are vectors of covariates with corresponding *α*_*j*_ vectors of regression coefficients, and *c* is the number of components. Odds ratios are calculated to assess the relationships between covariates and component membership.

Gamma mixture regression models allow estimation of component densities that depend on covariates and thus the covariates influence the mean LOS for each of the components. The Gamma mixture regression models relate the mean LOS to the covariates. For the *j*^*th*^ component, the mean LOS is modeled by a linear function of covariates via the log link.

$$\text{log}\;\left(\mu_{ji}\right)=\beta_{j0}+\beta_{j1}x_{i1}+\ldots +\beta_{jp}x_{ip}$$

where the *β*_*j*_ vectors are the regression coefficients that correspond to the *x*_*i*_ vectors of covariates. Mean ratios are calculated within each component to assess the relationship between covariates and LOS. Mean ratios compare the mean LOS in one level of a covariate compared to another level of the same covariate. For example, if the mean LOS among women aged 45 years and older was 6 and the mean LOS among women younger than 45 years was 3, then the mean ratio is 2.

### Modeling the effects of hospitals

The model defined above does not account for the fact that patients treated in the same hospital are correlated. The correlation of patients nested within hospitals may result in misleading inferences due to incorrect estimates of the standard errors if these effects are not incorporated into the LOS models [13]. The dependency of patients nested within hospitals can be accounted for by adding random hospital effects to the model. The random hospital effects are assumed to be independent and normally distributed. The random hospital effects capture the differences in clinical care and unmeasurable characteristics impacting the population in each hospital. Random effects specified in the component density functions affect the means of the components, and random effects specified in the mixing probabilities function affect the component memberships. Predicted random effects from fitting the different models provide estimates of inter-hospital variation adjusted for other factors.

### Comparison of Gamma mixture models

Four different model specifications are compared in this paper where the covariates and random effects are included in different portions of the model as summarized in Table 1.

**Table 1:** Placement of modeling effects in Gamma mixture models.

Model	Mixing Probabilities Function	Component Density
1	None	None
2	Covariates Hospital Effect	None
3	Covariates	Hospital Effect
4	None	Covariates Hospital Effect

Model 1) The LOS is fit to a Gamma mixture distribution with no covariates or random hospital effects. This serves as the baseline case for comparing models containing covariates and hospital effects. Model 1 is defined as:
  $$f(y_{i})=\sum_{j=1}^{c}\pi_{j}f_{j}\text{(}y_{i}\text{;}\theta_{j})$$
  where *f*_*j*_(*y*_*i*_;*θ*_*j*_) is the Gamma probability density function and *π*_*j*_ is the mixing probability for the *j*^*th*^ component.
Model 2) The mixing probabilities depend on covariates and random hospital effects. Model 2 is defined as:
  $$f(y_{i})=\sum_{j=1}^{c}\frac{exp\;(\sum_{l=0}^{p}\alpha_{jl}x_{il}+u_{k})}{1+\;\sum_{h=1}^{c-1}exp\;(\sum_{l=0}^{p}\alpha_{hl}x_{il}+u_{k})}f_{j}\text{(}y_{i}\text{;}\theta_{j})$$
  where the *x*_*i*_ are vectors of covariates with corresponding *a*_*j*_ vectors of regression coefficients, *u*_*k*_ is the random effect for the *k*^*th*^ hospital, *c* is the number of components, and *f*_*j*_(*y*_*i*_;*θ*_*j*_) is the Gamma probability density function for the *j*^*th*^ component.
Model 3) The mixing probabilities depend on covariates and random hospital effects are specified in the component densities. The density of Model 3 is defined as:
  $$f(y_{i})=\sum_{j=1}^{c}\frac{exp\;(\sum_{l=0}^{p}\alpha_{jl}x_{il})}{1+\;\sum_{h=1}^{c-1}\text{exp}\;\left(\sum_{l=0}^{p}\alpha_{hl}x_{il}\right)}f_{j}\text{(}y_{i}\text{;}\theta_{j}),\text{log}\;\left(\mu_{ji}\right)=u_{jk}$$
  where the *x*_*i*_ are vectors of covariates with corresponding *a*_*j*_ vectors of regression coefficients, *c* is the number of components, and *f*_*j*_(*y*_*i*_;*θ*_*j*_) is the Gamma probability density function with the mean (*μ*_*ji*_) modeled by the random effect for the *k*^*th*^ hospital (*u*_*jk*_) via the log link for the *j*^*th*^ component.
Model 4) The component density depends on both covariates and random hospital effects. Model 4 is defined as:
  $$f(y_{i})=\sum_{j=1}^{c}\pi_{j}f_{j}\text{(}y_{i}\text{;}\theta_{j}),\;\text{log}\;\left(\mu_{ij}\right)=\beta_{j0}+\beta_{j1}x_{i1}+\cdots +\beta_{jp}x_{ip}+u_{jk}$$
  where *f*_*j*_(*y*_*i*_;*θ*_*j*_) is the Gamma probability density function with the mean (*μ*_*ji*_) modeled by the *β*_*j*_ vectors of the regression coefficients that correspond to the *x*_*i*_ vectors of covariates and a random effect for the *k*^*th*^ hospital (*u*_*jk*_) via the log link, and *π*_*j*_ is the mixing probability for the *j*^*th*^ component.

### Estimation

In this paper the Gamma mixture regression models are fit using PROC NLMIXED in SAS version 9.4 (SAS Institute, Cary, NC). Example SAS code to fit the four Gamma mixture regression models is provided (S1 Appendix). We obtain maximum likelihood estimates for the unknown parameters in the models using integral approximations and numerical optimization algorithms [29]. The Adaptive Gaussian Quadrature method is used to fit the mixture regression model by providing an approximation of the likelihood integrated over the random effects [30]. The Dual Quasi-Newton optimization technique is used to perform the maximization [29]. The models without random hospital effects were fitted first to obtain good starting values for PROC NLMIXED. Good initial values are important to avoid non-convergence in the estimation. The maximum likelihood covariate coefficient estimates and standard errors are computed using the final Hessian matrix. The ratio of the covariate coefficient estimates and corresponding standard errors produce t-values and p-values calculated based on the t-distribution. The test for the random effects variance component should be interpreted with caution as the null hypothesis of the variance equals zero lies on the boundary of the parameter space. Thus, Empirical Bayes estimates are used to obtain predicted random hospital effects [29].

### Model goodness of fit

The models with different numbers of components are compared to each other based on the AIC statistical criteria. In using the AIC criteria for model comparison, the smaller AIC value indicates a better fit to the data. Model parameters were evaluated to ensure mixing probabilities were nonzero and that all components are different [22]. Model goodness of fit was evaluated through residual analyses and estimation of bias. Residuals were calculated as the difference between observed and predicted mean LOS for each covariate pattern. Bias was calculated as the average residual across all covariate patterns weighted by the size of the covariate pattern [31,32].

## Application

This study used New York State (NYS) hospital data from the New York Statewide Planning and Research Cooperative System (SPARCS) inpatient de-identified dataset for 2014 [33]. The dataset is publicly available on NYS Department of Health website and no Institutional Review Board approval is needed.

Records with All Patient Refined Diagnosis Related Groups codes (540, 560) for vaginal and Cesarean delivery were selected. Delivery hospitalizations were restricted to those discharged to home or self-care (96% of delivery hospitalizations). Patients transferred to other institutions are excluded since they would have additional LOS not measured in the unlinked file. Patients who died during their delivery hospitalization (11 women) are excluded as their true LOS had they survived is unknown.

The delivery LOS was defined as the number of days from admission to discharge. Patient characteristics of age, race/ethnicity, and primary insurance were included as potential covariates that influence LOS.

Hospitals’ teaching status and perinatal level of care were included as potential covariates that influence LOS. Teaching status (teaching hospital vs non-teaching hospital) was obtained from the Accreditation Council for Graduate Medical Education [34]. In New York, hospital perinatal levels of care are designated based on the hospital’s capabilities and types of health care providers available for care [35,36]. There are four perinatal levels of care; level 1 hospitals are only equipped for low risk deliveries, level 2 hospitals are equipped for deliveries with moderate risk, and levels 3 and 4 hospitals are equipped for high-risk deliveries [35]. Birthing facilities were stratified into level 1 or 2 hospitals and level 3 or 4 hospitals [4,37].

Table 2 summarizes patient and hospital characteristics by hospital location and delivery method. The distributions of characteristics differed by geographic region (New York City (NYC) vs rest of the state (ROS) hospitals) and by delivery method (vaginal vs Cesarean delivery). Table 2 also provides the mean LOS and standard deviation of LOS for the patient and hospital characteristics. To account for the interaction of hospital location with the other covariates, all models were stratified by geographic region (NYC and ROS).

**Table 2:** Patient and hospital characteristics by delivery method and region.

Characteristic	Vaginal Deliveries (n = 144,379)				Cesarean Deliveries (n = 73,076)			
	NYC^a^ (n = 74,738)		ROS^d^ (n = 69,641)		NYC (n = 35,778)		ROS (n = 37,298)	
	N (%)	Mean LOS^b^(SD^c^)	N (%)	Mean LOS(SD)	N (%)	Mean LOS(SD)	N (%)	Mean LOS(SD)
**Maternal Age**:								
Less than 30	37,473 (52.6%)	2.5 (1.3)	38,433 (55.2%)	2.4 (1.3)	13,152 (36.8%)	3.9 (2.6)	16,009 (42.9%)	3.6 (2.3)
30 and over	37,265 (49.9%)	2.4 (1.5)	31,208 (44.8%)	2.3 (1.3)	22,626 (63.2%)	3.9 (3.0)	21,289 (57.1%)	3.7 (3.0)
**Race/ethnicity**:								
Black, non-Hispanic	12,293 (16.4%)	2.7 (1.9)	7,541 (10.8%)	2.5 (1.4)	7,553 (21.1%)	4.2 (3.0)	4,470 (12.0%)	4.1 (3.2)
Hispanic	14,615 (19.6%)	2.6 (1.4)	7,557 (10.9%)	2.4 (0.9)	7,237 (20.2%)	3.9 (2.4)	4,275 (11.5%)	3.8 (2.8)
Other, non-Hispanic	22,787 (30.5%)	2.5 (1.2)	9,416 (13.5%)	2.4 (1.4)	11,469 (32.1%)	3.8 (2.8)	5,055 (13.6%)	4.0 (3.0)
White, non-Hispanic	25,043 (33.5%)	2.3 (1.3)	45,127 (64.8%)	2.3 (1.3)	9,519 (26.6%)	3.8 (3.1)	23,498 (63.0%)	3.5 (2.5)
**Primary Insurance**:								
Medicaid	46,256 (61.9%)	2.5 (1.4)	32,029 (46.0%)	2.4 (1.3)	20,235 (56.6%)	3.9 (2.7)	15,362 (41.2%)	3.7 (2.8)
Private	28,419 (38.1%)	2.4 (1.5)	37,603 (54.0%)	2.3 (1.3)	15,509 (43.4%)	3.9 (3.2)	21,924 (58.8%)	3.7 (2.6)
**Hospital Level**:								
Levels 1,2	7,597 (10.2%)	2.5 (0.8)	39,251 (56.4%)	2.3 (0.7)	3,933 (11.0%)	3.5 (1.1)	19,452 (52.2%)	3.3 (1.1)
Levels 3,4	67,140 (89.8%)	2.5 (1.5)	30,387 (43.6%)	2.5 (1.8)	31,845 (89.0%)	4.0 (3.0)	17,845 (47.8%)	4.1 (3.7)
**Teaching Status**:								
Yes	50,385 (67.4%)	2.4 (1.5)	22,309 (32.0%)	2.5 (1.6)	24,098 (67.4%)	4.0 (3.2)	13,392 (35.9%)	4.1 (3.5)
No	24,353 (32.6%)	2.5 (1.3)	47,332 (68.0%)	2.3 (1.1)	11,680 (32.6%)	3.8 (2.2)	23,906 (64.1%)	3.5 (2.1)
**Length of Stay**								
1 day	2,720 (3.6%)		4,953 (7.1%)		30 (0.1%)		115 (0.3%)	
2–3 days	67,798 (90.7%)		61,250 (88.0%)		19,582 (54.7%)		21,566 (57.8%)	
4–6 days	3,738 (5.0%)		3,095 (4.4%)		14,802 (41.4%)		14,572 (39.1%)	
7–14 days	354 (0.5%)		246 (0.4%)		1,028 (2.9%)		763 (2.1%)	
15+ days	128 (0.2%)		96 (0.1%)		336 (0.9%)		281 (0.8%)	

^a^ New York City.

^b^ Length of Stay.

^c^ Standard deviation.

^d^ Rest of State (New York State excluding New York City).

Gamma mixture regression models were fitted to delivery hospitalization LOS by delivery method using patient and hospital covariates. For each stratum (delivery method by hospital location), the data was initially investigated without restriction on the number of components (subpopulations). For efficiency of space, the results for NYC Cesarean deliveries are provided here, and the results for NYC vaginal deliveries, ROS vaginal deliveries, and ROS Cesarean deliveries are in the supplemental tables (S1–S6 Tables).

Throughout all analyses two components provided the best fit, with component A capturing common LOS and component B capturing long LOS and one day stays. The one day stays (0.08%) have a negligible impact on the results.

### Model 1: No covariates

When no covariates are considered, a two component Gamma mixture model was found to best fit the empirical distribution of LOS for NYC Cesarean deliveries. Component A captured LOS ranging from 2 to 6 days (median = 3 days), that is the common LOSs for uncomplicated Cesarean deliveries. Component B predominately captured longer LOS ranging from 7 to 97 days (median = 9 days). The model fit could not be enhanced by adding more components as the model was nonidentifiable due to overfitting (fitting too many components).

### Developing models to measure association between covariates and LOS

Patient and hospital covariates are next added to the Gamma mixture models in the mixing probabilities function or component density to assess their influence on LOS. Since labor and delivery procedures within a hospital impact all women delivering at that hospital, random effects need to be included in the analyses. The results were consistent across hospital locations and types of delivery (S1–S6 Tables). The associations of teaching status, age, and primary insurance with LOS differed across each stratum. All models were consistent in identifying the longer LOSs occurred in hospitals designated to care for the most complex patients (levels 3 and 4).

To assess the association between covariates and longer LOS, we estimated the odds of a women’s hospitalization being assigned to component B (longer stays) compared to component A (common LOS) when the covariates were placed in the mixing probabilities function (Model 2 and Model 3). We estimated mean ratios for LOS in each component when the covariates were placed in the component densities (Model 4).

### Model 2: Covariates and random hospital effects in the mixing probabilities function

When patient and hospital covariates and random hospital effects were included, a two-component Gamma mixture regression model was found to provide the best model fit to the data (Table 3). Each woman was assigned to either component A or component B based the maximum of their posterior probabilities. Component A generally captured women with shorter LOS and accounts for an estimated 95% of the NYC Cesarean deliveries with LOSs ranging from 2 to 6 days. Component B accounts for an estimated 5% of the NYC Cesarean deliveries with the majority LOSs ranging from 6 to 97 days.

**Table 3:** Model 2 covariates and random hospital effects in the mixing probabilities function for belonging to component B for NYC Cesarean deliveries.

Covariates	Reference Category	Logistic Parameter Estimate (S.E.)
**Intercept**		-4.43 (0.32)*
**Maternal Age**: 30 and over	Under 30	0.04 (0.06)
**Race/Ethnicity**:		
Black, non-Hispanic	White, non-Hispanic	0.68 (0.09)*
Hispanic		0.05 (0.10)
Other, non-Hispanic		-0.03 (0.08)
**Primary Insurance**: Medicaid	Private	0.31 (0.07)*
**Hospital Level**: Levels 3,4	Levels 1,2	1.40 (0.32)*
**Teaching Status**: Yes	No	0.05 (0.22)
**Variance component**		0.59*
**AIC**	98863	

* p-value < 0.05.

The AIC statistic improved from Model 1 with no covariates (AIC = 99584.1) to Model 2 with covariates and random hospital effects in the mixing probabilities function (AIC = 98863).

The odds of belonging to component B for Black, non-Hispanic women are 1.98 (95% CI: 1.62–2.33) times the odds for White, non-Hispanic women adjusted for all other covariates. Women delivering in level 3 or 4 hospitals are associated with longer LOS compared to women delivering in level 1 or 2 hospitals (Table 4).

**Table 4:** Adjusted odds ratios (OR) with 95% confidence intervals for component B membership for NYC Cesarean sections.

Covariate	Model 2	Model 3
	OR (95% CI)	OR (95% CI)
**Maternal Age**:		
30 and over vs Under 30	1.04 (0.92–1.17)	1.03 (0.91–1.15)
**Race/Ethnicity**:		
Black, Non-Hispanic vs White, Non-Hispanic	1.98 (1.62–2.33)	2.25 (1.88–2.62)
Hispanic vs White, Non-Hispanic	1.05 (0.84–1.25)	1.31 (1.06–1.55)
Other, Non-Hispanic vs White, Non-Hispanic	0.97 (0.80–1.14)	1.01 (0.84–1.18)
**Primary Insurance**:		
Medicaid vs Private	1.37 (1.17–1.57)	1.35 (1.18–1.53)
**Hospital Level**:		
Levels 3,4 vs Levels 1,2	4.03 (1.42–6.66)	2.66 (1.84–3.48)
**Teaching Status**:		
Yes vs No	1.05 (0.59–1.50)	0.99 (0.86–1.12)

**Fig 2:**
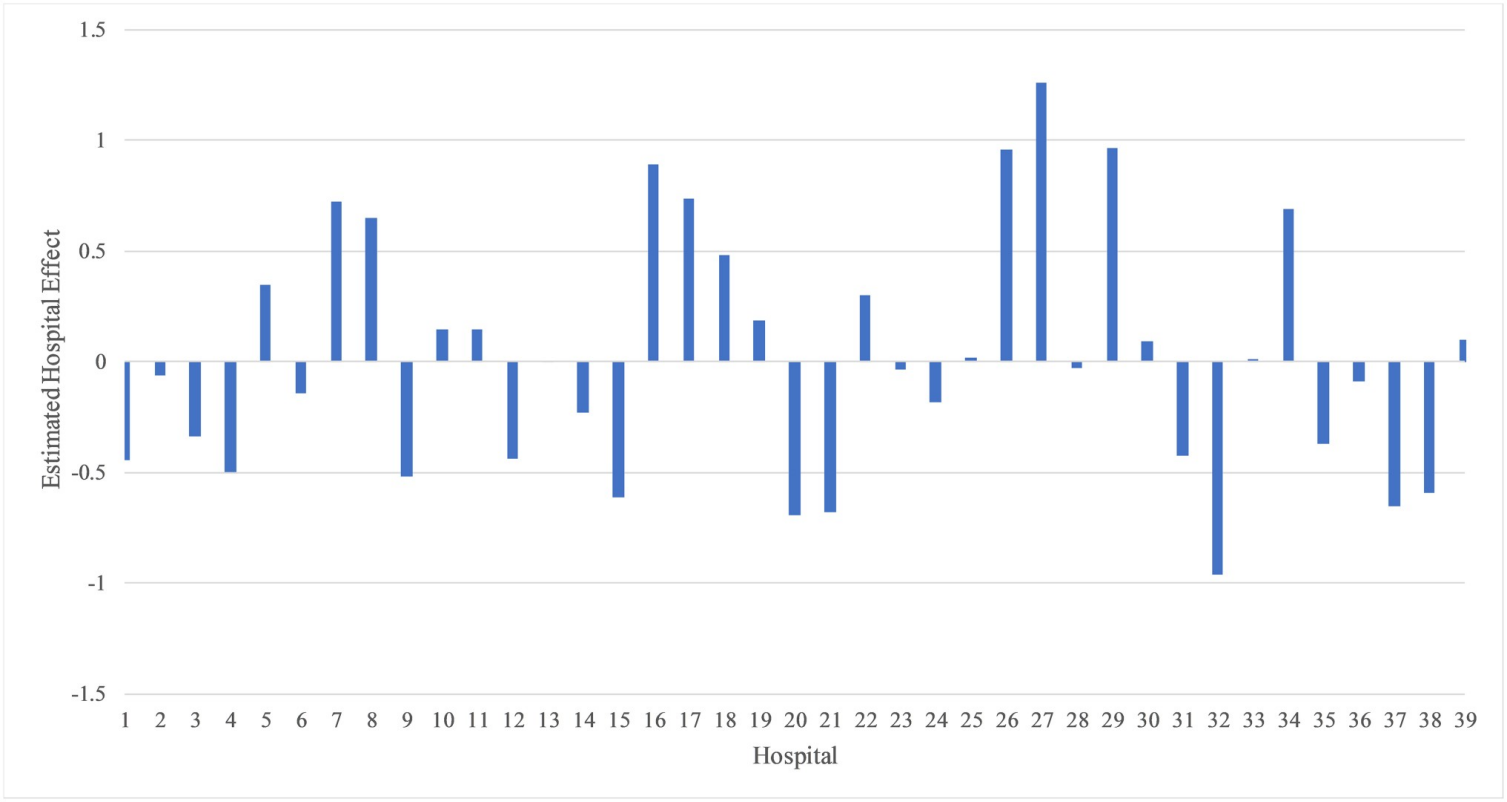
Estimated hospital effects from fitting Model 2 with covariates and random hospital effects in the mixing probabilities function for belonging to component B for New York City Cesarean deliveries.

Fig 2 shows the predicted random hospital effects for assignment to component B (longer LOS). Hospital 27 has the largest effect of women more likely to belong to component B. Hospital 27 is a level 4 teaching hospital with 61% of the deliveries covered by private insurance and 72% of the women who delivered aged 30 years and older. Hospital 32 has the largest effect of women more likely to belong to component A (shorter LOS). Hospital 32 is a level 3 teaching hospital with a high proportion (90%) of women covered by Medicaid.

### Model 3: Covariates in the mixing probabilities function and random hospital effects in the Gamma regression

Component A accounts for an estimated 95% and component B accounts for an estimated 5% of the NYC Cesarean deliveries. The estimated component LOS ranges are the same as in Model 2. The AIC statistic improved from Model 2 (AIC = 98863) to Model 3 with covariates in the mixing probabilities function and random hospital effects in the Gamma regression (AIC = 97020).

Black, non-Hispanic race/ethnicity, hospital level, and primary insurance have the same associations for component membership in both Model 2 and Model 3. The odds of belonging to component B (longer LOS) for women with Medicaid as primary insurance are 1.35 (95% CI: 1.20–1.53) times the odds of women with private primary insurance adjusted for all other covariates (Tables 4 and 5).

**Table 5:** Model 3 covariates in the mixing probabilities function for belonging to component B and random hospital effects in the Gamma regression for NYC Cesarean deliveries.

Covariates	Reference Category	Logistic Parameter Estimate (S.E.)	Comp A	Comp B
**Intercept**		-3.90 (0.18)*		
**Maternal Age**: 30 and over	Under 30	0.03 (0.06)		
**Race/Ethnicity**:				
Black, Non-Hispanic	White, Non-Hispanic	0.81 (0.08)*		
Hispanic		0.27 (0.09)*		
Other, Non-Hispanic		0.01 (0.08)		
**Primary Insurance**: Medicaid	Private	0.30 (0.06)*		
**Hospital Level**: Levels 3,4	Levels 1,2	0.98 (0.15)*		
**Teaching Status**: Yes	No	-0.01 (0.06)		
**Variance component**			0.002*	0.07*
**AIC**	97020			

* p-value < 0.05.

**Fig 3:**
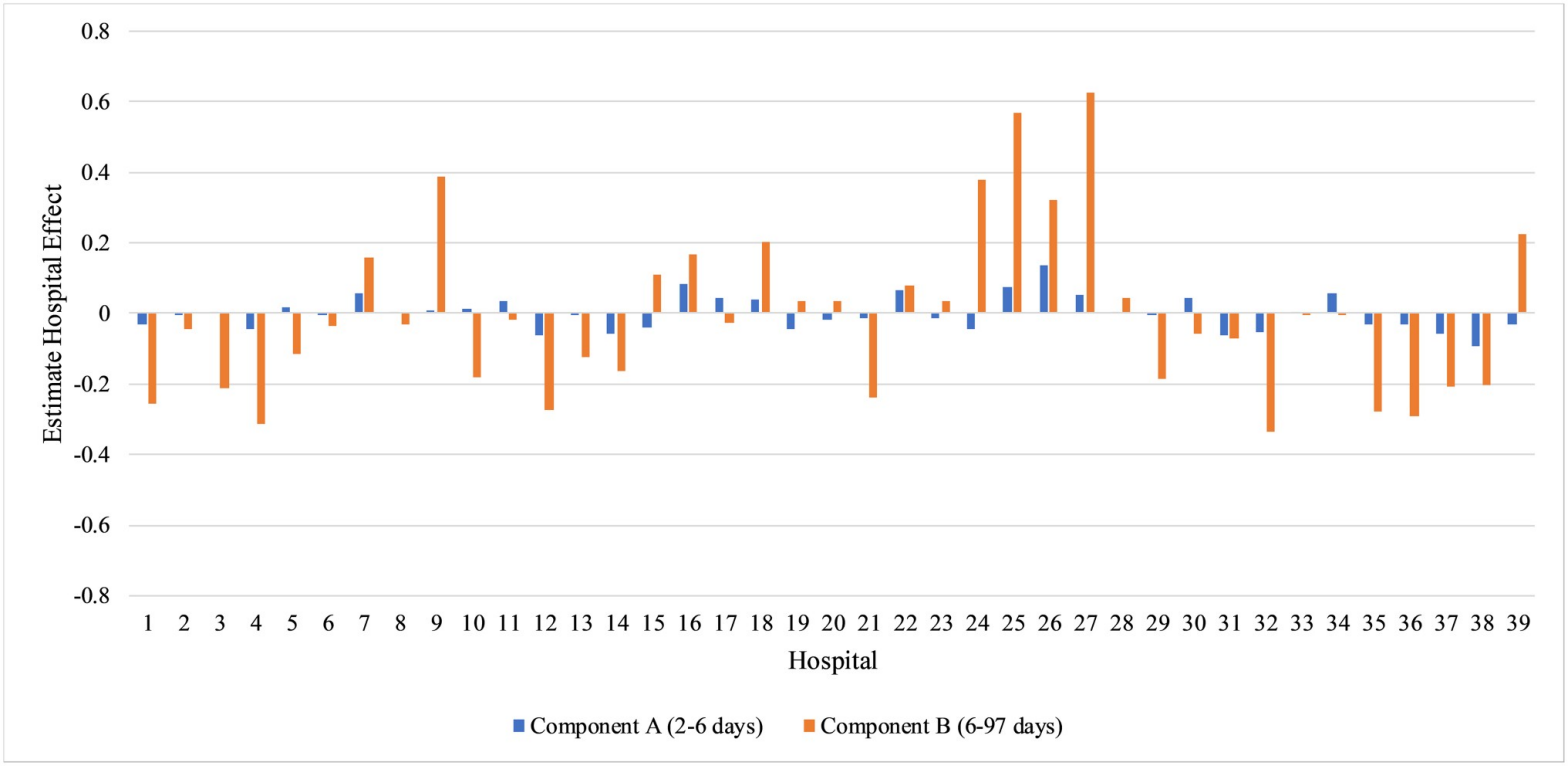
Estimated hospital effects from fitting Model 3 with covariates in the mixing probabilities function for belonging to component B and random hospital effects in the Gamma regression for New York City Cesarean deliveries.

For component A, the performance of the hospitals are similar and no large random effect predictions are found (Fig 3). For component B, Hospitals 25 and 27 have the largest effects on lengthening the LOS, and Hospitals 4 and 32 have the largest effects on shortening the LOS. Hospitals 25 is a large level 4 teaching hospital with a high proportion (92%) of women with private insurance and Hospital 4 is a smaller level 3 non-teaching hospital with a 94% of women covered by Medicaid.

### Model 4: Covariates and random hospital effects in the Gamma regression

Component A accounts for an estimated 95% of the NYC Cesarean deliveries with LOS ranging from 2 to 7 days. Component B accounts for an estimated 5% of the NYC Cesarean deliveries with LOS ranging from 6 to 97 days and includes the one-day stays. The AIC statistic improved from Model 2 (AIC = 98863) and Model 3 (AIC = 97020) to Model 4 with covariates and random hospital effects in the Gamma regression (AIC = 96829). The differences in the AIC statistic for the models with covariates were small.

**Fig 4:**
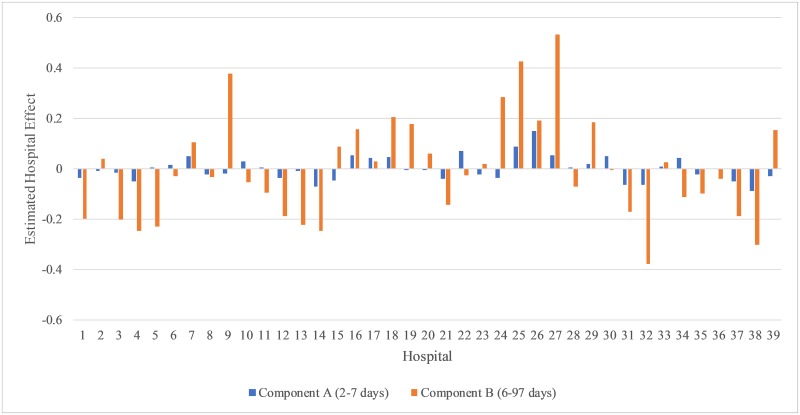
Estimated hospital effects from fitting Model 4 with covariates and random hospital effects in the Gamma regression for New York City Cesarean deliveries.

The set of significant covariates that influence LOS for each component (subpopulation) differ. The magnitude of the parameter estimates and standard errors are larger for component B (Table 6). Among women’s LOSs in component A (LOSs between 2 and 6 days for NYC Cesarean deliveries), maternal age and race are the statistically significant covariates. The mean LOS for women aged thirty and older is 0.97 (95% CI: 0.96–0.97) times the mean LOS for women under the age of thirty, adjusted for all other covariates (Table 7). Black, non-Hispanic women are associated with longer stays compared to White, non-Hispanic women. Specifically, the mean LOS for Black, non-Hispanic women is 1.06 (95% CI: 1.05–1.07) times the mean LOS for White, non-Hispanic women in component A, adjusted for all other covariates. This can also be interpreted as a 6% increase in the mean LOS for Black, non-Hispanic women compared to White, non-Hispanic women. The performance of the hospitals are similar and no large random effect predictions are found (Fig 4).

**Table 6:** Model 4 covariates and random hospital effects in the Gamma regression for NYC Cesarean deliveries.

Covariate	Reference Category	Component A Estimate (S.E.)	Component B Estimate (S.E.)
**Intercept**		1.22 (0.02)*	1.46 (0.12)*
**Maternal Age**: 30 and over	Under 30	-0.03 (0.003)*	0.04 (0.03)
**Race/Ethnicity**:			
Black, Non-Hispanic	White, Non-Hispanic	0.06 (0.004)*	0.17 (0.05)*
Hispanic		0.01 (0.004)	-0.02 (0.05)
Other, Non-Hispanic		0.01 (0.003)*	0.06 (0.04)
**Primary Insurance**:			
Medicaid	Private	0.001 (0.003)	0.08 (0.04)*
**Hospital Level**: Levels 3,4	Levels 1,2	0.03 (0.02)	0.48 (0.12)*
**Teaching Status**: Yes	No	0.01 (0.02)	0.13 (0.09)
**Mixing probability**		0.93	
**Variance component**		0.002*	0.05*
**AIC**	96829		

* p-value < 0.05

**Table 7:** Adjusted mean ratios (MR) with 95% confidence intervals for length of stay for Model 4 for NYC Cesarean sections.

Covariate	Component A	Component B
	MR (95% CI)	MR (95% CI)
**Maternal Age**:		
30 and over vs Under 30	0.97 (0.96–0.97)	1.04 (0.97–1.11)
**Race/Ethnicity**:		
Black, Non-Hispanic vs White, Non-Hispanic	1.06 (1.05–1.07)	1.18 (1.06–1.30)
Hispanic vs White, Non-Hispanic	1.01 (1.00–1.02)	0.98 (0.88–1.08)
Other, Non-Hispanic vs White, Non-Hispanic	1.01 (1.00–1.02)	1.07 (0.97–1.16)
**Primary Insurance**:		
Medicaid vs Private	1.00 (0.99–1.01)	1.08 (1.01–1.16)
**Hospital Level**:		
Levels 3,4 vs Levels 1,2	1.03 (0.99–1.01)	1.62 (1.23–2.00)
**Teaching Status**:		
Yes vs No	1.01 (0.98–1.04)	1.14 (0.94–1.33)

Among women’s LOSs in component B (LOSs primarily between 7 and 97 days for NYC Cesarean deliveries), race, primary insurance, and hospital level are the statistically significant covariates. On average, Black, non-Hispanic women had longer stays compared to White, non-Hispanic women. Specifically, the mean LOS for Black, non-Hispanic women is 1.18 (95% CI: 1.06–1.30) times the mean LOS for White, non-Hispanic women in component B, adjusted for all other covariates. Women delivering in level 3 and 4 hospitals are associated with longer stays in component B compared to women delivering in level 1 and 2 hospitals (Table 7). Hospitals 25 and 27 have the largest effects on lengthening the LOS, and Hospital 32 has the largest effect on shortening the LOS (Fig 4).

Overall, the AIC statistic improved substantially from Model 1 to all the models with covariates and random hospital effects.

## Discussion

As research on SMM increases in the effort to improve maternal outcomes, the need for good statistical models for the entire LOS distribution, including the tail of very long LOSs, is imperative. Gamma mixture models are particularly useful for research on LOS. These models can analyze the complete LOS data distribution and are relatively robust to investigator decisions. For example, regardless of the placement of the covariates and random hospital effects, the models predict the vast majority of women to the same components (subpopulations). Specifically, for NYC Cesarean deliveries all models that included covariates predicted 97.9% of the women to the same component. Of the 2.1% of women with a predicted component change in at least one model, all had LOS of 6 or 7 days (the boundary region of LOS between the two component distributions (common LOS and long LOS)).

Overall, the observed differences in LOS for NYC Cesarean deliveries for all three models with covariates and random hospital effects are associated with race, primary insurance, and hospital level. Model 2 and 3 provide insight into covariates associated with having a longer LOS through odds ratios of being assigned to component B. Model 4 provides information about how covariates are associated with lengthening LOS within each component (long and short stay distributions) through mean ratios for comparing mean LOS by covariate levels.

Covariates found to be associated with LOS are similar across regions for Cesarean deliveries. Associated covariates with LOS vary for vaginal deliveries across regions. For example, age is associated with longer LOS for NYC vaginal deliveries but not for ROS vaginal deliveries (S1–S6 Tables). Fewer covariates influence LOS for vaginal deliveries compared to Cesarean deliveries. This is not surprising given the differences in the delivery processes, increased variability in LOS for each method, and the additional risks associated with Cesarean delivery [38].

A limitation of using NYS public use de-identified discharge data is that comorbidities are not available. Future studies to incorporate comorbidities would move the field forward. Studying comorbidities is complicated as some occur prior to hospitalization, useful for predicting LOS, and others during hospitalization. Unfortunately, large national databases do not provide the nuanced information needed to fully sort out when comorbidities occur. The patient and hospital characteristics available in the data are known before admission, this allows for prediction of LOS before the delivery hospitalization. The hospital variation in component membership and LOS is significant in all the models, indicating unexplained differences in LOS among hospitals after adjusting for the available covariates; hospitals have effects beyond the covariates measured. Diagnosis codes in conjunction with present on admission codes could be used to identify comorbidities (e.g. hypertension) that could vary between hospitals and explain part of the observed hospital effects. However, when comorbidities are included, hospital effects are notable when studying SMM and risk for Cesarean delivery [4,37,38]. After obtaining and evaluating these additional risk factors, hospital protocols should be reviewed to understand differences in care not captured by measured factors.

Finite mixture regression models can be complex and computationally intensive to implement. The unknown parameters in finite mixture modeling can be estimated using numerical methods or EM algorithms. EM algorithms are widely used and numerically stable. However, EM algorithms do not estimate the variance-covariance matrix. The variance-covariance matrix is necessary to calculate the standard errors and the tests of statistical significance for the estimated parameters. We use an alternative numerical method of Dual Quasi-Newton optimization to estimate the unknown parameters and to provide the variance-covariance matrix. Results of finite mixture models estimated with numerical methods and EM algorithms have been found to be comparable [17].

While some studies used covariates to model both the mixing probabilities and component density means, we did not fit this specification of a finite mixture regression to avoid identifiability problems [21,39]. In such complex and computationally intensive models, using a large number of parameters tends to overtax the models and they do not converge.

The values of residuals for all models for NYC Cesarean deliveries ranged from -0.43 to 0.55 days. There was very good agreement between the averages of the observed and predicted lengths of stay within each covariate pattern with smaller differences in the covariate patterns with a large number of patients (over 100). The largest difference between observed and predicted lengths of stays for Model 2, Model 3 and Model 4 was in a covariate pattern with only 3 patients. The model biases for the three models were less than two tenths of a day and the positive biases represent underestimation of the models by approximately one tenth of a day in predicting mean LOS (Table 8). We are using NYS publicly available de-identified administrative data and thus are limited in the covariate choices. The limitation in the choice of covariates and not being able to include health status information likely lead to the underestimation of the mean LOS. Individual patient comorbidities are unlikely to substantially change prediction of components (shorter LOS and longer LOS distributions) [40,41]. As with modeling in general the effect of a parameter changes depending on the other covariates in the model. Changing the covariates in finite mixture regression models influences both the component membership and parameter effects.

**Table 8:** Bias of the models for NYC Cesarean deliveries.

Model	Bias
2	0.12
3	0.11
4	0.12

Thirty (0.08%) women who had Cesarean delivery in NYC had LOSs of one day recorded in the administrative data. It is most likely that these LOSs are errors [42]. One day stays were assigned to component B (longer hospital stays) however they are irrelevant in the analyses. When the 30 women were removed from the analyses, the findings were not importantly changed.

This study shows the predictive power of including covariates in modeling LOS for delivery hospitalizations. The different placement of covariates and random effects produced consistent results. Future work will focus on selecting important covariates for application in finite mixture regression models for LOS.

## Supporting information

S1 Appendix SAS code for fitting Gamma mixture models. (DOCX)

S1 Table Model 1 Gamma mixture model with no covariates. (DOC)

S2 Table Model 2 covariates and random hospital effects in the mixing probabilities function for belonging to component B. (DOC)

S3 Table Model 3 covariates in the mixing probabilities function for belonging to component B and random hospital effects in the Gamma regression. (DOC)

S4 Table Adjusted odds ratios (OR) with 95% confidence intervals for component B membership. (DOC)

S5 Table Model 4 covariates and random hospital effects in the Gamma regression. (DOC)

S6 Table Adjusted mean ratios (MR) with 95% confidence intervals for length of stay. (DOC)

## References

[1] Papanicolas I, Woskie LR, Jha AK. Health Care Spending in the United States and Other High-Income Countries. JAMA. 2018;31910: 1024–39. 10.1001/jama.2018.1150 29536101

[2] Geller SE, Koch AR, Garland CE, MacDonald EJ, Storey F, Lawton B. A global view of severe maternal morbidity: moving beyond maternal mortality. Reprod Health. 2018;15(Suppl 1): 98 10.1186/s12978-018-0527-2 29945657 PMC6019990

[3] Callaghan W, Creanga A, Kuklina E. Severe maternal morbidity among delivery hospitalizations in the United States. Obstet Gynecol. 2012;120(5):1029–1036. 10.1097/AOG.0b013e31826d60c5 23090519

[4] Lazariu V, Nguyen T, McNutt LA, Jeffrey J, Kacica M. Severe maternal morbidity: A population-based study of an expanded measure and associated factors. PLoS One. 2017;12(8):e0182343 10.1371/journal.pone.0182343 28787028 PMC5546569

[5] McDermott KW, Elixhauser A, Sun R. Trends in Hospital Inpatient Stays in the United States, 2005–2014. HCUP Statistical Brief #225. June 2017. Agency for Healthcare Research and Quality, Rockville, MD. https://www.hcup-us.ahrq.gov/reports/statbriefs/sb225-Inpatient-US-StaysTrends.pdf

[6] Center for Medicare and Medicaid Services. Newborns' and Mothers' Health Protection Act (NMHPA). May 4, 2013. https://www.cms.gov/CCIIO/Programs-and-Initiatives/Other-Insurance-Protections/nmhpa_factsheet.html.

[7] Hospital Inpatient National Statisitics, 2014. HCUPnet. Healthcare Cost and Utilization Project. Agency for Healthcare Research and Quality, Rockville, MD. https://hcupnet.ahrq.gov

[8] Lu MC. Reducing Maternal Mortality in the United States. JAMA. 2018;320(12): 1237–8. 10.1001/jama.2018.11652 30208484

[9] Lee AH, Xiao J, Vemuri SR, Zhao Y. A discordancy test approach to identify outliers of length of hospital stay. Stat Med. 1998;17(19): 2199–206. 10.1002/(sici)1097-0258(19981015)17:19<2199::aid-sim917>3.0.co;2-2 9802178

[10] Lee AH, Gracey M, Wang K, Yau KK. A robustified modeling approach to analyze pediatric length of stay. Ann Epidemiol. 2005;15(9): 673–7. 10.1016/j.annepidem.2004.10.001 16157254

[11] Wang K, Yau KK, Lee AH. A hierarchical Poisson mixture regression model to analyse maternity length of hospital stay. Stat Med. 2002;21(23): 3639–54. 10.1002/sim.1307 12436461

[12] Lee AH, Fung WK, Fu B. Analyzing hospital length of stay: mean or median regression? Med Care. 2003;41(5): 681–6. 10.1097/01.MLR.0000062550.23101.6F 12719692

[13] Leung KM, Elashoff RM, Rees KS, Hasan MM, Legorreta AP. Hospital- and patient-related characteristics determining maternity length of stay: a hierarchical linear model approach. Am J Public Health. 1998;88(3): 377–81. 10.2105/ajph.88.3.377 9518967 PMC1508339

[14] Cots F, Elvira D, Castells X, Saez M. Relevance of outlier cases in case mix systems and evaluation of trimming methods. Health Care Manag Sci. 2003;6(1): 27–35. 10.1023/a:1021908220013 12638924

[15] Freitas A, Silva-Costa T, Lopes F, Garcia-Lema I, Teixeira-Pinto A, Brazdil P, et al Factors influencing hospital high length of stay outliers. BMC Health Serv Res. 2012;12: 265 10.1186/1472-6963-12-265 22906386 PMC3470984

[16] Marazzi A, Paccaud F, Ruffieux C, Beguin C. Fitting the distributions of length of stay by parametric models. Med Care. 1998;36(6): 915–27. 10.1097/00005650-199806000-00014 9630132

[17] Lee AH, Wang K, Yau KK, McLachlan GJ, Ng SK. Maternity length of stay modelling by gamma mixture regression with random effects. Biom J. 2007;49(5): 750–64. 10.1002/bimj.200610371 17722201

[18] Faddy M, Graves N, Pettitt A. Modeling length of stay in hospital and other right skewed data: comparison of phase-type, gamma and log-normal distributions. Value Health. 2009;12(2): 309–14. 10.1111/j.1524-4733.2008.00421.x 20667062

[19] Ng Sk, Yau KKW, Lee AH. Modelling Inpatient Length of Stay by a Hierarchical Mixture Regression via the EM Algorithm. Mathematical and Computer Modeling. 2003;37: 365–75.

[20] Xiao J, Lee AH, Vemuri SR. Mixture distribution analysis of length of hospital stay for efficient funding. Socio-Economic Planning Sciences. 1999;33(1): 39–59.

[21] Yau KKW, Lee AH, Ng ASK. Finite mixture regression model with random effects: application to neonatal hospital length of stay. Computational Statistics & Data Analysis. 2003;41: 359–66.

[22] McLachlan G, Peel D. Finite Mixture Models. New York: John Wiley & Sons; 2000.

[23] Lee AH, Ng AS, Yau KK. Determinants of maternity length of stay: a Gamma mixture risk-adjusted model. Health Care Manag Sci. 2001;4(4): 249–55. 10.1023/a:1011810326113 11718457

[24] Wang K, Yau KK, Lee AH. A zero-inflated Poisson mixed model to analyze diagnosis related groups with majority of same-day hospital stays. Comput Methods Programs Biomed. 2002;68(3): 195–203. 10.1016/s0169-2607(01)00171-7 12074846

[25] Lee AH, Xiao J, Codde JP, Ng AS. Public versus private hospital maternity length of stay: a gamma mixture modelling approach. Health Serv Manage Res. 2002;15(1): 46–54. 10.1258/0951484021912824 11854995

[26] Singh CH, Ladusingh L. Inpatient length of stay: a finite mixture modeling analysis. Eur J Health Econ. 2010;11(2): 119–26. 10.1007/s10198-009-0153-6 19430985

[27] Quantin C, Sauleau E, Bolard P, Mousson C, Kerkri M, Brunet Lecomte P, et al Modeling of high-cost patient distribution within renal failure diagnosis related group. J Clin Epidemiol. 1999;52(3): 251–8. 10.1016/s0895-4356(98)00164-4 10210243

[28] Ickowicz A, Sparks R. Modelling hospital length of stay using convolutive mixtures distributions. Stat Med. 2017;36(1): 122–35. 10.1002/sim.7135 27704639

[29] SAS Institute Inc. 2011. SAS/STAT^®^ 9.3 User’s Guide. Cary, NC: SAS Institute Inc.

[30] Pinheiro J, Bates DM. Approximations to the Log-Likelihood Function in the Nonlinear Mixed-Effects Model. J Comput Graph Stat. 1995;4(1): 12–35.

[31] Liu C, Zhang L, Davis CJ, Solomon D, Gove J. A Finite Mixure Model for Characterizing the Diameter Distributions of Mixed-Specied Forest Stands. Forest Science. 2002;48(4):653–61.

[32] Verburg IW, de Keizer NF, de Jonge E, Peek N. Comparison of regression methods for modeling intensive care length of stay. PLoS One. 2014;9(10):e109684 10.1371/journal.pone.0109684 25360612 PMC4215850

[33] New York State Department of Health. Hospital Inpatient Discharges (SPARCS De-Identified) 2014. https://health.data.ny.gov/Health/Hospital-Inpatient-Discharges-SPARCS-De-Identified/rmwa-zns4.

[34] Accreditation Council for Graduate Medical Education. 2018. https://www.acgme.org/.

[35] New York State Department of Health. Perinatal Regionalization. 2018. https://www.health.ny.gov/community/pregnancy/health_care/perinatal/regionalization_descrip.htm

[36] New York Sate Department of Health. NYS Health Profiles: 17 Regional Perinatal Centers in New York. https://profiles.health.ny.gov/hospital/designated_center/Regional+Perinatal+Center

[37] Howell EA, Zeitlin J, Hebert PL, Balbierz A, Egorova N. Association between hospital-level obstetric quality indicators and maternal and neonatal morbidity. JAMA. 2014;312(15): 1531–41. 10.1001/jama.2014.13381 25321908 PMC4334152

[38] Kozhimannil KB, Arcaya MC, Subramanian SV. Maternal Clinical Diagnoses and Hospital Variation in the Risk of Cesarean Delivery: Analyses of a National US Hospital Discharge Database. PLoS Med. 2014;11(10):e1001745 10.1371/journal.pmed.1001745 25333943 PMC4205118

[39] Thompson TJ, Smith PJ, Boyle JP. Finite mixture models with concomitant information: assessing diagnostic criteria for diabetes. J R Stat Soc Ser C Appl Stat. 1998;47(3): 393–404.

[40] Mulla ZD, Nuwayhid BS, Garcia KM, Flood-Shaffer K, Van Hook JW, Hampton RM. Risk factors for a prolonged length of stay in women hospitalized for preeclampsia in Texas. Hypertens Pregnancy. 2010;29(1): 54–68. 10.3109/10641950902777754 19909212

[41] Blumenfeld YJ, El-Sayed YY, Lyell DJ, Nelson LM, Butwick AJ. Risk Factors for Prolonged Postpartum Length of Stay Following Cesarean Delivery. Am J Perinatol. 2015;32(9): 825–32. 10.1055/s-0034-1543953 25594218 PMC4504826

[42] Main EK, Abreo A, McNulty J, Gilbert W, McNally C, Poeltler D, et al Measuring severe maternal morbidity: validation of potential measures. Am J Obstet Gynecol. 2016;214(5): 643.e1-.e10. 26582168 10.1016/j.ajog.2015.11.004

