# Supplementary material for: Dealing with highly skewed hospital length of stay distributions: The use of Gamma mixture models to study delivery hospitalizations
Source: PLoS One. 2020 Apr 20;15(4):e0231825. doi: 10.1371/journal.pone.0231825 (PMC7170466; doi:10.1371/journal.pone.0231825)
Supplement: S1 Appendix — (DOCX) [file pone.0231825.s001.docx]

/*SAS code for fitting Gamma mixture models for New York City Cesarean deliveries.

Initial values of the parameters are specified by using the PARMS statement. */

* Model 1: No covariates;

ODS graphics on;

**PROC** **FMM** DATA=nyc_cesarean PLOTS=ALL COMPONENTINFO FITDETAILS;

MODEL length_of_stay =/ DIST=gamma k=**2**;

OUTPUT out=max class=ML;

**RUN**;

*Model 2: Covariates and random hospital effects in the mixing probabilities function;

**PROC** **NLMIXED** DATA=nyc_cesarean GCONV=**0**;

BOUNDS nu1>**0**, nu2>**0**, s1>**0**, s2>**0**;

PARMS x1=**1.2556** x2=**2.2221** nu1=**22.4066** nu2=**2.0792** b10=-**4.3981** b11=**0.07702** b12=**0.7593** b13=**0.2249** b14=-**0.02008** b15=**0.2235** b16=**1.4061** b17=**0.1065** s1=**0.5** s2=**0.5**;

mu1 = exp(x1+u1);

mu2 = exp(x2+u2);

ka1 = (nu1*Length_of_Stay) / (mu1);

ka2 = (nu2*Length_of_Stay) / (mu2);

f1 = ((ka1**nu1)*exp(-ka1)) / (Length_of_Stay*gamma(nu1));

f2 = ((ka2**nu2)*exp(-ka2)) / (Length_of_Stay*gamma(nu2));

p = exp(b10 + b11*old + b12*black + b13*hisp + b14*other + b15*medi + b16*level34 + b17*yesteach)/(**1**+exp(b10 + b11*old + b12*black + b13*hisp + b14*other + b15*medi + b16*level34 + b17*yesteach));

ll = log((**1**-p)*f1 + p*f2);

MODEL Length_of_Stay ~ general(ll);

RANDOM u1 u2 ~ normal([**0**,**0**],[s1,**0**,s2]) subject = facility_id;

PREDICT ((**1**-p)*f1)/((**1**-p)*f1 + p*f2) OUT=predp1(rename=(pred=postp1));

PREDICT (p*f2)/((**1**-p)*f1 + p*f2) OUT=predp2(rename=(pred=postp2));

**RUN**;

*Model 3: Covariates in the mixing probabilities function and random hospital effects in the Gamma regression ;

**PROC** **NLMIXED** DATA=nyc_cesarean GCONV=**0**;

BOUNDS nu1>**0**, nu2>**0**, s1>**0** ;

PARMS x1=**1.2556** x2=**2.2221** nu1=**22.4066** nu2=**2.0792** b10=-**4.3981** b11=**0.07702** b12=**0.7593** b13=**0.2249** b14=-**0.02008** b15=**0.2235** b16=**1.4061** b17=**0.1065** s1=**0.5** ;

mu1 = exp(x1);

mu2 = exp(x2);

ka1 = (nu1*Length_of_Stay) / (mu1);

ka2 = (nu2*Length_of_Stay) / (mu2);

f1 = ((ka1**nu1)*exp(-ka1)) / (Length_of_Stay*gamma(nu1));

f2 = ((ka2**nu2)*exp(-ka2)) / (Length_of_Stay*gamma(nu2));

p = exp(b10 + b11*old + b12*black + b13*hisp + b14*other + b15*medi + b16*level34 + b17*yesteach +u1)/(**1**+exp(b10 + b11*old + b12*black + b13*hisp + b14*other + b15*medi + b16*level34 + b17*yesteach + u1));

ll = log((**1**-p)*f1 + p*f2);

MODEL Length_of_Stay ~ general(ll);

RANDOM u1 ~ normal(**0**,s1*s1) subject = facility_id;

PREDICT ((**1**-p)*f1)/((**1**-p)*f1 + p*f2) OUT=predp1(rename=(pred=postp1));

PREDICT (p*f2)/((**1**-p)*f1 + p*f2) OUT=predp2(rename=(pred=postp2));

**RUN**;

*Model 4: Covariates and random hospital effects in the Gamma regression;

**PROC** **NLMIXED** DATA=nyc_cesarean GCONV=**0**;

BOUNDS s1>**0**, s2>**0**, nu1>**0**, nu2>**0**;

PARMS nu1=**22.6082** nu2=**2.1315** b10=**1.2265** b11=-**0.02779** b12=**0.05180** b13=**0.01478** b14=**0.008393** b15=-**0.02121** b16=**0.04143** b17=**0.009428**

b20=**1.5867** b21=**0.04208** b22=**0.09253** b23=**0.000874** b24=**0.05658** b25=**0.000589** b26=**0.5023** b27=**0.1421** s1=**0.5** s2=**0.5** x1=**2.5952**;

mu1 = exp(b10 +b11*old + b12*black + b13*hisp + b14*other + b15*medi + b16*level34 + b17*yesteach + u1);

mu2 = exp(b20 + b21*old + b22*black + b23*hisp + b24*other + b25*medi + b26*level34 + b27*yesteach + u2);

ka1 = (nu1*length_of_stay) / (mu1);

ka2 = (nu2*length_of_stay) / (mu2);

f1 = ((ka1**nu1)*exp(-ka1)) / (length_of_stay*gamma(nu1));

f2 = ((ka2**nu2)*exp(-ka2)) / (length_of_stay*gamma(nu2));

p1 = exp(x1)/(**1**+exp(x1));

ll = log(p1*f1 + (**1**-p1)*f2 );

MODEL length_of_stay ~ general(ll);

RANDOM u1 u2~ normal([**0**,**0**],[s1,**0**,s2]) subject = facility_id;

PREDICT (p1*f1)/(p1*f1 + (**1**-p1)*f2) OUT=predp1(rename=(pred=postp1));

PREDICT ((**1**-p1)*f2)/(p1*f1 + (**1**-p1)*f2 OUT=predp2(rename=(pred=postp2));

**RUN**;
