# Supplementary material for: Dealing with highly skewed hospital length of stay distributions: The use of Gamma mixture models to study delivery hospitalizations
Source: PLoS One. 2020 Apr 20;15(4):e0231825. doi: 10.1371/journal.pone.0231825 (PMC7170466; doi:10.1371/journal.pone.0231825)
Supplement: S1 Table — (DOC) [file pone.0231825.s002.doc]

|  | **NYC^a^ Vaginal Deliveries** | **ROS^b^ Vaginal Deliveries** | | **ROS Cesarean Deliveries** | |
| --- | --- | --- | --- | --- | --- |
| **Parameter** | **Estimate** | **Comp A Estimate** | **Comp B**  **Estimate** | **Comp A Estimate** | **Comp B Estimate** |
| **Mean** | 2.47 | 2.29 | 2.61 | 3.41 | 10.35 |
| **Shape** | 8.73 | 26.80 | 2.66 | 14.24 | 1.46 |
| **Mixing probability** | 1.00 | 0.77 | 0.23 | 0.96 | 0.04 |
| **AIC** | 179403 | 146341 | | 109471 | |

^a^ New York City

^b^ Rest of State (New York State excluding New York City
