# Supplementary material for: Dealing with highly skewed hospital length of stay distributions: The use of Gamma mixture models to study delivery hospitalizations
Source: PLoS One. 2020 Apr 20;15(4):e0231825. doi: 10.1371/journal.pone.0231825 (PMC7170466; doi:10.1371/journal.pone.0231825)
Supplement: S2 Table — (DOC) [file pone.0231825.s003.doc]

|  | | **NYC^a^ Vaginal Deliveries** | **ROS^b^ Vaginal Deliveries** | **ROS Cesarean Deliveries** |
| --- | --- | --- | --- | --- |
| **Covariate** | **Reference Category** | **Logistic Parameter Estimate (S.E.)** | **Logistic Parameter Estimate (S.E.)** | **Logistic Parameter Estimate (S.E.)** |
| **Intercept** |  | -2.46 (0.29)* | -5.54 (0.25)* | -5.60 (0.26)* |
| **Maternal Age**: 30 and over | Under 30 | 0.13 (0.04)* | -0.10 (0.09) | 0.04 (0.08) |
| **Race/ethnicity**:  Black, Non-Hispanic  Hispanic  Other, Non-Hispanic | White, Non-Hispanic | -0.23 (0.06)*  -0.33 (0.06)*  -0.31 (0.05)* | 0.16 (0.12)  -0.01 (0.15)  -0.17 (0.13) | 0.66 (0.10)*  0.15 (0.13)  0.05 (0.11) |
| **Primary Insurance**: Medicaid | Private | -0.30 (0.05)* | 0.10 (0.10) | 0.28 (0.09)* |
| **Hospital Level**: Levels 3,4 | Levels 1,2 | 0.64 (0.31)* | 1.63 (0.38)* | 2.03 (0.36)* |
| **Teaching Status**: Yes | No | 0.03 (0.23) | 0.55 (0.43) | 0.62 (0.39) |
| **Variance component** |  | 0.63* | 1.14* | 1.01* |
| **AIC** |  | 152403 | 147264 | 108482 |

*p-value < 0.05

^a^ New York City

^b^ Rest of State (New York State excluding New York City)
