# Supplementary material for: Dealing with highly skewed hospital length of stay distributions: The use of Gamma mixture models to study delivery hospitalizations
Source: PLoS One. 2020 Apr 20;15(4):e0231825. doi: 10.1371/journal.pone.0231825 (PMC7170466; doi:10.1371/journal.pone.0231825)
Supplement: S3 Table — (DOC) [file pone.0231825.s004.doc]

|  | | **NYC^a^ Vaginal Deliveries** | | | **ROS^b^ Vaginal Deliveries** | | | **ROS Cesarean Deliveries** | | |
| --- | --- | --- | --- | --- | --- | --- | --- | --- | --- | --- |
| **Covariate** | **Reference Category** | **Logistic Parameter Estimate (S.E.)** | **Comp**  **A** | **Comp**  **B** | **Logistic Parameter Estimate (S.E.)** | **Comp**  **A** | **Comp**  **B** | **Logistic Parameter Estimate (S.E.)** | **Comp**  **A** | **Comp**  **B** |
| **Intercept** |  | -2.73 (0.12)* |  | | -0.92 (0.03)* |  | | -2.90 (0.10)* |  | |
| **Maternal Age**: 30 and over | Under 30 | 0.20 (0.04)* |  |  | -0.08 (0.05)* |  |  | -0.22 (0.07)* |  |  |
| **Race/ethnicity**:  Black, NH^c^  Hispanic  Other, NH | White, NH | -0.22 (0.06)*  -0.39 (0.06)*  -0.44 (0.05)* |  |  | -0.33 (0.05)*  -0.67 (0.06)*  -0.54 (0.05)* |  |  | 0.27 (0.08)*  -0.49 (0.11)*  -0.33 (0.10)* |  |  |
| **Primary Insurance**: Medicaid | Private | -0.50 (0.04)* |  |  | 0.08 (0.03)* |  |  | 0.44 (0.09)* |  |  |
| **Hospital Level**: Levels 3,4 | Levels 1,2 | 0.64 (0.10)* |  |  | -0.03 (0.04) |  |  | 0.46 (0.08)* |  |  |
| **Teaching Status**: Yes | No | 0.49 (0.05)* |  |  | -0.26 (0.04)* |  |  | -0.20 (0.08)* |  |  |
| **Variance component** |  |  | 0.002* | 0.11* |  | 0.001* | 0.10* |  | 0.02* | 0.23* |
| **AIC** |  | 149788 | | | 141823 | | | 101852 | | |

*p-value < 0.05

^a^ New York City

^b^ Rest of State (New York State excluding New York City)

^c^ Non-Hispanic
