# Supplementary material for: Dealing with highly skewed hospital length of stay distributions: The use of Gamma mixture models to study delivery hospitalizations
Source: PLoS One. 2020 Apr 20;15(4):e0231825. doi: 10.1371/journal.pone.0231825 (PMC7170466; doi:10.1371/journal.pone.0231825)
Supplement: S4 Table — (DOC) [file pone.0231825.s005.doc]

|  | **NYC^a^ Vaginal Deliveries** | | **ROS^b^ Vaginal Deliveries** | | **ROS Cesarean Deliveries** | |
| --- | --- | --- | --- | --- | --- | --- |
| **Covariate** | **Model 2**  **OR (95% CI)** | **Model 3**  **OR (95% CI)** | **Model 2**  **OR (95% CI)** | **Model 3**  **OR (95% CI)** | **Model 2**  **OR (95% CI)** | **Model 3**  **OR (95% CI)** |
| **Maternal Age**:  30 and over vs Under 30 | 1.14 (1.04-1.23) | 1.22 (1.12-1.32) | 0.90 (0.75-1.06) | 0.93 (0.87-0.98) | 1.04 (0.88-1.21) | 0.81 (0.70-0.91) |
| **Race/Ethnicity:**  Black, NH^c^ vs White, NH  Hispanic vs White, NH  Other, NH vs White, NH | 0.80 (0.70-0.90)  0.72 (0.63-0.81)  0.73 (0.66-0.81) | 0.80 (0.71-0.89)  0.68 (0.60-0.76)  0.64 (0.58-0.70) | 1.18 (0.90-1.45)  0.99 (0.69-1.29)  0.85 (0.63-1.06) | 0.72 (0.65-0.79)  0.52 (0.46-0.58)  0.59 (0.53-0.64) | 1.94 (1.55-2.33)  1.16 (0.85-1.48)  1.05 (0.81-1.29) | 1.31 (1.10-1.53)  0.61 (0.47-0.75)  0.72 (0.58-0.85) |
| **Primary Insurance**:  Medicaid vs Private | 0.74 (0.67-0.81) | 0.61 (0.56-0.66) | 1.10 (0.89-1.31) | 1.08 (1.01-1.15) | 1.33 (1.09-1.56) | 1.56 (1.35-1.77) |
| **Hospital Level**:  Levels 3,4 vs Levels 1,2 | 1.90 (0.72-3.07) | 1.89 (1.50-2.28) | 5.08 (1.23-8.93) | 0.97 (0.90-1.04) | 7.62 (2.21-13.02) | 1.59 (1.29-1.88) |
| **Teaching Status**:  Yes vs No | 1.03 (0.56-1.50) | 1.64 (1.47-1.81) | 1.73 (0.24-3.24) | 0.77 (0.71-0.83) | 1.85 (0.40-3.30) | 0.82 (0.68-0.95) |

^a^ New York City

^b^ Rest of State (New York State excluding New York City

^c^ Non-Hispanic
