# Supplementary material for: Dealing with highly skewed hospital length of stay distributions: The use of Gamma mixture models to study delivery hospitalizations
Source: PLoS One. 2020 Apr 20;15(4):e0231825. doi: 10.1371/journal.pone.0231825 (PMC7170466; doi:10.1371/journal.pone.0231825)
Supplement: S5 Table — (DOC) [file pone.0231825.s006.doc]

|  |  | **NYC^a^ Vaginal Deliveries** | | **ROS^b^ Vaginal Deliveries** | | **ROS Cesarean Deliveries** | |
| --- | --- | --- | --- | --- | --- | --- | --- |
| **Covariate** | **Reference Category** | **Comp A Estimate (S.E.)** | **Comp B Estimate (S.E.)** | **Comp A Estimate (S.E.)** | **Comp B**  **Estimate (S.E.)** | **Comp A Estimate (S.E.)** | **Comp B Estimate (S.E.)** |
| **Intercept** |  | 0.89 (0.01)* | 0.81 (0.10)* | 0.79 (0.01)* | 0.93 (0.06)* | 1.12 (0.02)* | 1.34 (0.05)* |
| **Maternal Age**: 30 and over | Under 30 | -0.02 (0.002)* | 0.02 (0.02) | -0.03 (0.002)* | -0.01 (0.04) | -0.03 (0.003)* | 0.07 (0.03)* |
| **Race/ethnicity:**  Black, NH^c^  Hispanic  Other, NH | White, NH | 0.04 (0.003)*  0.03 (0.003)*  0.02 (0.003)* | 0.51 (0.03)*  0.33 (0.03)*  0.23 (0.02)* | 0.03 (0.004)*  0.02 (0.004)*  0.002 (0.004) | 0.15 (0.06)*  0.02 (0.06)  0.05 (0.06) | 0.04 (0.005)*  0.01 (0.005)  0.03 (0.004)* | 0.13 (0.04)*  0.12 (0.05)*  0.10 (0.05)** |
| **Primary Insurance**: Medicaid | Private | -0.01 (0.003)* | 0.13 (0.02)* | -0.01 (0.003)* | 0.06 (0.04) | -0.01 (0.003)* | -0.05 (0.03) |
| **Hospital Level**: Levels 3,4 | Levels 1,2 | -0.01 (0.02) | 0.14 (0.10) | 0.08 (0.02)* | 0.79 (0.10)* | 0.12 (0.04)* | 0.74 (0.08)* |
| **Teaching Status**: Yes | No | -0.01 (0.01) | -0.07 (0.07) | 0.03 (0.03) | 0.16 (0.11) | 0.06 (0.04) | 0.31 (0.10)* |
| **Variance component** |  | 0.001* | 0.05* | 0.007* | 0.08* | 0.01* | 0.06* |
| **Mixing probability** |  | 0.89 |  | 0.97 |  | 0.94 |  |
| **AIC** |  | 149831 | | 144508 | | 139636 | |

*p-value < 0.05

**p-value=0.0513

^a^ New York City

^b^ Rest of State (New York State excluding New York City)

^c^ Non-Hispanic
