# Supplementary material for: Dealing with highly skewed hospital length of stay distributions: The use of Gamma mixture models to study delivery hospitalizations
Source: PLoS One. 2020 Apr 20;15(4):e0231825. doi: 10.1371/journal.pone.0231825 (PMC7170466; doi:10.1371/journal.pone.0231825)
Supplement: S6 Table — (DOC) [file pone.0231825.s007.doc]

|  | **NYC^a^ Vaginal Deliveries** | | **ROS^b^ Vaginal Deliveries** | | **ROS Cesarean Deliveries** | |
| --- | --- | --- | --- | --- | --- | --- |
| **Covariate** | **Component A**  **MR (95% CI)** | **Component B**  **MR (95% CI)** | **Component A**  **MR (95% CI)** | **Component B**  **MR (95% CI)** | **Component A**  **MR (95% CI)** | **Component B**  **MR (95% CI)** |
| **Maternal Age**:  30 and over vs Under 30 | 0.98 (0.98-0.99) | 1.02 (0.98-1.05) | 0.97 (0.96-0.97) | 0.99 (0.91-1.06) | 0.97 (0.97-0.98) | 1.08 (1.01-1.14) |
| **Race/Ethnicity:**  Black, NH^c^ vs White, NH  Hispanic vs White, NH  Other, NH vs White, NH | 1.04 (1.03-1.05)  1.03 (1.02-1.03)  1.02 (1.02-1.03) | 1.67 (1.57-1.77)  1.39 (1.31-1.47)  1.25 (1.20-1.77) | 1.03 (1.02-1.04)  1.02 (1.01-1.02)  1.00 (0.99-1.01) | 1.17 (1.03-1.30)  1.02 (0.88-1.17)  1.05 (0.92-1.17) | 1.05 (1.04-1.06)  1.01 (1.00-1.02)  1.04 (1.03-1.04) | 1.14 (1.04-1.24)  1.13 (1.01-1.25)  1.10 (0.99-1.21) |
| **Primary Insurance**:  Medicaid vs Private | 0.99 (0.98-0.99) | 1.14 (1.10-1.19) | 0.99 (0.98-0.99) | 1.07 (0.98-1.15) | 0.99 (0.98-0.99) | 0.96 (0.89-1.02) |
| **Hospital Level**:  Levels 3,4 vs Levels 1,2 | 0.99 (0.95-1.02) | 1.15 (0.91-1.38) | 1.08 (1.03-1.13) | 2.21 (1.78-2.64) | 1.13 (1.05-1.21) | 2.10 (1.76-2.45) |
| **Teaching Status**:  Yes vs No | 0.99 (0.95-1.02) | 0.93 (0.79-1.07) | 1.04 (0.97-1.10) | 1.17 (0.91-1.42) | 1.07 (0.97-1.16) | 1.37 (1.11-1.63) |

^a^ New York City

^b^ Rest of State (New York State excluding New York City

^c^ Non-Hispanic
